# Comprehensive Study of the Enzymatic Catalysis of the Electrochemical Oxygen Reduction Reaction (ORR) by Immobilized Copper Efflux Oxidase (CueO) From *Escherichia coli*

**DOI:** 10.3389/fchem.2018.00358

**Published:** 2018-08-24

**Authors:** Sara Chumillas, Beatriz Maestro, Juan M. Feliu, Víctor Climent

**Affiliations:** ^1^Institute of Electrochemistry, University of Alicante, Alicante, Spain; ^2^Instituto de Biología Molecular y Celular, Universidad Miguel Hernández, Elche, Spain

**Keywords:** bioelectrocatalysis, CueO, protein voltammetry, oxygen reduction reaction, laccase

## Abstract

In recent years, enzymatic fuel cells have experienced a great development promoted by the availability of novel biological techniques that allow the access to a large number of enzymatic catalysts. One of the most important aspects in this area is the development of biocatalysts for the oxygen reduction reaction (ORR). Laccases from the group of enzymes called blue multi-cooper oxidases have received considerable attention because of their ability to catalyze the electrochemical oxygen reduction reaction to water when immobilized on metallic or carbonaceous electrode materials. In this paper we report a comprehensive study of the electrocatalytic activity of the enzyme Copper efflux oxidase (CueO) from *Escherichia coli* immobilized on different electrode materials. The influence of the electrode substrate employed for protein immobilization was evaluated using glassy carbon, gold or platinum electrodes. Gold and platinum electrodes were modified using different self-assembled monolayers (SAM) able to tune the electrostatic interaction between the protein and the substrate, depending on the nature of the terminal functional group in the SAM. The effects of protein immobilization time, electrode potential, solution pH and temperature, protein and O_2_ concentration have been carefully investigated. Finally, direct electron transfer (DET) was investigated in the presence of the following inhibitors: fluoride (F^−^), chloride (Cl^−^) and azide (${\text{N}}_{3}^{-}$).

## Introduction

Significant efforts have been devoted to the investigation and development of biofuel cells (Barton et al., 2004; Heller, 2004; Minteer et al., 2007; Cooney et al., 2008; Moehlenbrock and Minteer, 2008; Willner et al., 2009; Luckarift et al., 2014; Hickey et al., 2016; Rasmussen et al., 2016; Ruzgas, 2017). The main motivation for this research is to replace the catalysts typically present in conventional fuel cells, often based on expensive noble metals such as platinum, with enzymatic or related bioinspired catalysts based on much more available first row transition metals. The development of new enzymatic catalysts for the oxygen (O_2_) reduction to water (H_2_O) in cathodes of biofuel cells is one of the main goals sought by different research groups working in this field (Barton et al., 2004; Heller, 2004; Minteer et al., 2007; Cooney et al., 2008; Cracknell et al., 2008; Moehlenbrock and Minteer, 2008; Willner et al., 2009; Luckarift et al., 2014). For this purpose, laccases and, more generally, blue copper oxidases, have received considerable attention because of their ability to catalyze the electrochemical O_2_ reduction when immobilized on metallic or carbonaceous electrodes (Gupta et al., 2004; Shleev et al., 2005a,b, 2006; Blanford et al., 2007; Klis et al., 2007; Tominaga et al., 2008; Murata et al., 2009; Schubert et al., 2009; Dos Santos et al., 2010; Ivnitski et al., 2010; Climent et al., 2012, 2014; Dagys et al., 2014; Pankratov et al., 2014; Adam et al., 2016; Di Bari et al., 2016; Bogdanovskaya et al., 2017; Jamshidinia et al., 2017). It has been shown that laccases, or related enzymes, can be as effective as platinum, with an onset for the oxygen reduction reaction (ORR) similar to the one found in this metal, but cheaper to prepare or to find in nature (Mano et al., 2003; Dos Santos et al., 2010).

The natural activity of the enzymes of the group called blue multi-copper oxidases (MCOs) couples the oxidation of organic substrates with the reduction of O_2_ to H_2_O (Solomon et al., 1996, 2008; Lee et al., 2002; Morozova et al., 2007; Sakurai and Kataoka, 2007). Enzymes of this family possess homologous structures, with two actives sites formed by one and three copper atoms (Solomon et al., 1996, 2008; Lee et al., 2002; Morozova et al., 2007; Sakurai and Kataoka, 2007). The first active site, called type 1 (*Cu*_*T*1_), is where the substrate oxidation takes place. This is the entry point of electrons to the protein. The second active center is where the O_2_ reduction takes place. This site consists of a trinuclear copper cluster, which is formed by a type 2 copper atom and a type 3 copper dimer. The electrons from the oxidation of the substrate are transported through a chain of aminoacids (His-Cys-His) from the type 1 copper atom to the type 2/3 copper cluster, where they combine with O_2_, producing its reduction to H_2_O.

When immobilized on the electrode, this one replaces the natural substrate of the enzyme as electron donor, while the type 2/3 copper cluster still is able to reduce O_2_ to water. To achieve effective catalysis of the ORR, enzyme immobilization must achieve a correct orientation on the electrode, leaving the type 2/3 copper cluster exposed to the solution, without changing its three-dimensional structure, while favoring fast rates of electron transfer between the electrode and the protein (Climent et al., 2012, 2014; Vaz-Dominguez et al., 2012).

The aim of this work is to determine the best experimental conditions (potential, immobilization time, temperature, pH, oxygen and protein concentration) to fabricate electrochemical devices with the ability to catalyze the ORR based on the bacterial laccase CueO (copper efflux oxidase from *Escherichia coli*). Although fungal laccases are normally selected for the catalysis of ORR, given their higher redox potential, their prokaryotic counterparts have several advantages. The main ones derive from the possibility of being cloned and overexpressed in *E. coli*, allowing the use of easy purification methodologies. Moreover, prokaryotic oxidases usually exhibit higher thermostability and operate in a wider pH range in comparison with the fungal enzymes (Santhanam et al., 2011; Martins et al., 2015).

While CueO conserves the structure common to all laccases, described above, it differs from it by containing a fifth weakly bonded copper atom. This atom plays a catalytic role in the protein and it is coordinated by two aspartates (Asp), two methionines (Met) and a H_2_O molecule (Roberts et al., 2002, 2003). Further crystallographic studies revealed that the fifth copper atom is bonded to the N-terminal of a methionine-rich region near the Cu_T1_ and also near the binding site of the substrate (Roberts et al., 2002).

Only a few articles describe CueO behavior on carbon black (Tsujimura et al., 2008; Kontani et al., 2009; Miura et al., 2009), pyrolitic graphite (Miura et al., 2007), or gold (Climent et al., 2014; Sugimoto et al., 2015). In this work we obtain new information about CueO immobilization on different electrode materials through a comprehensive analysis of the different factors that affect the protein performance and stability. These include length of immobilization time, temperature, pH, potential, oxygen and protein concentration. The role of the substrate is investigated by comparing the behavior on gold (Au) single crystal or polycrystalline electrode, polycrystalline platinum (Pt) and glassy carbon (GC). Finally, in order to obtain more information about the pathways of electron transfer from the supporting electrode to the protein, CueO catalytic activity for the ORR was evaluated in the presence of different inhibitors: sodium fluoride (NaF), sodium chloride (NaCl) and sodium azide (NaN_3_).

## Experimental

### Materials and reagents

4-aminothiophenol (4-ATP or pATP), cysteamine (CYS), cystamine, L-cysteine, mercaptopropionic acid (MPA), mercaptoundecanoic acid (MUA), 4-mercaptobenzoic acid (4-MB), hexanethiol (HT), imidazole, 2,6-dimethoxyphenol (DMP), NHS (N-hydroxysuccinimide), EDC (1-(3-dimethylaminopropyl)-3-ethylcarbodiimide hydrochloride), Na_2_HPO_4_, NaH_2_PO_4_H_2_O, and sodium chloride (NaCl) were obtained from Sigma-Aldrich. Sodium fluoride (NaF) and sodium azide (NaN_3_) were obtained from Merck-Suprapur. All chemicals were used as received without further purification.

### Purification of CueO protein

CueO purification and protein characterization measurements were done following the same procedure as in ref (Climent et al., 2014). Protein specific activity was measured following spectroscopically the oxidation of DMP as described previously (Climent et al., 2014). Resulting values were between 0.2 and 0.5 U mg^−1^, where one unit of activity (U) is defined as the amount of protein able to oxidize 1 μmol of DMP in 1 min at pH = 6.5 and at a temperature of 37°C.

### Electrode preparation

Before starting experiments, the glassy carbon electrode was polished with alumina 0.3 μm, rinsed with ultrapure water, immersed in a vial with water and sonicated for 5 min approximately.

The gold and platinum electrode surfaces were cleaned by heating in a butane flame immediately before its introduction in the supporting electrolyte solution or prior to the thiol modification. Self-Assembled monolayer (SAM) modification on gold and platinum electrodes was achieved following the same procedure indicated in reference (Xie et al., 2001). SAMs of MPA, cysteamine, cystamine and L-cysteine were prepared by immersing the electrode in 1 mM thiol aqueous solutions. On the other hand, MUA, 4-ATP, 4-MB and hexanethiol SAMs were created immersing the electrode in 1 mM solutions in ethanol of the corresponding thiols.

### Protein immobilization

In all cases, blank responses of modified and unmodified electrodes for the O_2_ reduction were registered initially in the absence of protein. Then, the protein was immobilized on the electrode surface by immersing it in 50 μL of the protein solution in 50 mM phosphate buffer, 100 mM NaCl, pH 7.0, during 5–10 min. After this time, the electrode was taken out from the solution and rinsed with 100 μL of the same phosphate buffer working solution to eliminate non-immobilized protein molecules. Finally, the electrode was immersed in the experimental cell and the voltammetric experiment started.

Covalent immobilization was performed by activation of carboxylate groups on both MPA SAM or protein groups using NHS/EDC mixed solutions of different concentrations. Then, protein-enzyme covalent binding was performed after electrode immersion in the corresponding protein sample (in case of MPA SAM activation groups) or after immersion of the cysteamine or 4-ATP modified electrode into the activated protein solution.

### Experimental setup

Electrochemical measurements were performed using a three-electrode electrochemical set up. As working electrodes, Au(111) basal plane and glassy carbon were used while reference and counter electrodes were a Ag/AgCl/saturated KCl and a gold wire, respectively. To carry out the measurements, a μAutolab type III was used as a potentiostat. The electrolytic medium (pH 6.5) was composed of a phosphate buffer solution (PBS) as supporting electrolyte, containing a mixture of NaH_2_PO_4_ and Na_2_HPO_4_ in ultrapure water (Ultra Elga PURELAB, 18.2 MΩ cm).

All experiments were performed at room temperature (ca. 25°C) except for the study of the effect of the temperature on enzyme activity. In this case, the temperature of the solution was controlled by immersing the electrochemical cell in a thermostatic bath controlled by a PolyScience digital temperature controller. The pH of each employed solution was measured using a Crison 507 pH meter. Experiments at different pH were done by adjusting the pH of each solution by adding perchloric acid (HClO_4_) and sodium hydroxide (NaOH). Initially the phosphate buffer solution was oxygenated by bubbling oxygen (O_2_) and measurements were performed under oxygen atmosphere. Experiments with different O_2_ concentration were performed using a combination of two gas flow controllers (Smart-Trak), to mix argon (Ar) and O_2_ in different proportions while keeping a constant flow rate of 10 scc/s for all the measurements.

## Results

**Figure 1:**
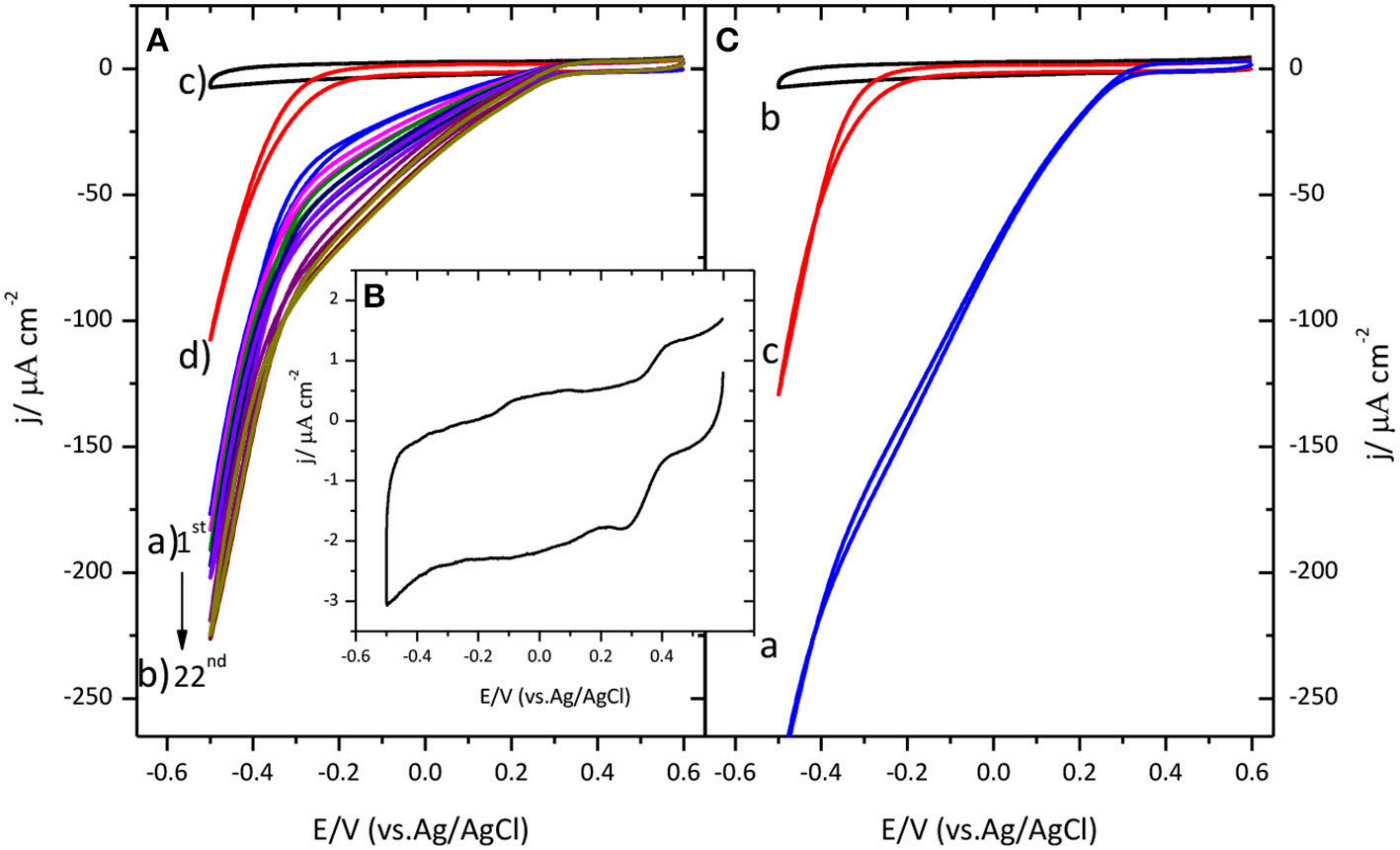
**(A)** Cyclic voltammogram for CueO immobilized on bare GC upon cycling in an O_2_ saturated sodium PBS, pH 6.5. (a) Blue line: 1st cycle. (b) Dark yellow line: 22nd cycle. (The arrow indicates the evolution from the first to the 22nd cycle). (c) GC blank in Ar atmosphere (black line). (d) GC blank in O_2_ atmosphere (Red line). **(B)** Cyclic voltammogram for CueO immobilized on bare GC in an Ar PBS, pH 6.5, after 22 cycles. **(C)** (a) First cyclic voltammetric curve for CueO (activated in Ar) in an O_2_ saturated PBS, pH 6.5, blue line: 1st cycle. (b) GC blank in Ar atmosphere (black line). (c) GC blank in O_2_ atmosphere (red line). Scan rate: 20 mVs^−1^.

Figure 1A shows the distinctive voltammetric profile for the catalysis of the ORR by immobilized CueO on a glassy carbon electrode.

Prior to CueO immobilization, the voltammogram characteristic of the glassy carbon substrate was recorded in Ar and O_2_ saturated buffer solutions. Comparison of the results obtained in the presence and in the absence of the protein reveals a displacement of the onset of the ORR by about 550 mV toward more positive values when protein is present on the electrode surface. An increase of the catalytic reduction current density is recorded during the initial 15 cycles. After those initial cycles, the magnitude of the current density remains nearly constant. It will be shown below that similar activation also happens when the protein is immobilized on other surfaces. Similar activation process was also reported for a related enzyme, the laccase from *Streptomyces coelicolor* (Climent et al., 2012), although no explanation was given in this case for this phenomenon.

To better understand the activation process that takes place during the initial potential cycles after protein immobilization, the following experiments were performed. The aim is to elucidate whether the initial activation observed in Figure 1A is related with the protein itself or with a modification of the carbon substrate that could happen, for instance, if new active groups are created on its surface during potential cycling that could favor electron transfer. To test this possibility, CueO was immobilized on a glassy carbon electrode that had been subjected, prior to the protein immobilization, to 22 potential cycles in an oxygen saturated phosphate buffer solution. When the protein is subsequently added to the surface, an increase of the current density is observed as before during the initial cycles (the result is shown in Supporting Information, Figure S1). If the evolution of the current were due to an activation of the carbon surface, such activation would also take place during the potential cycling in the absence of the protein and the maximum catalytic current would be observed in the first cycle after protein immobilization. Since this is not the case, this experiment allows us to exclude the activation of the carbon (formation of new functional groups) as the reason for the initial current increase and points toward a modification in the protein adlayer as the origin of this behavior. Such modification is most likely a reorientation of the protein at the electrode surface toward a shorter distance between the active site of the enzyme and the electrode surface.

Secondly, to determine the influence of oxygen in solution on this activation process, immobilized CueO on glassy carbon was exposed to several potential cycles in an argon saturated solution. The results are collected in Figure 1B, showing the appearance of a redox couple at 0.27/0.42 V when potential cycling in argon atmosphere. The average peak potential for this redox process is 0.35 V, a value that is in accordance with the reported value for the formal potential of the CueO type 1 copper site (*E*^∘^_T1_) (Tsujimura et al., 2008; Miura et al., 2009). After extensive potential cycling with the immobilized protein, the solution was bubbled with O_2_ until complete saturation. In this case, there is no current density increase upon cycling, and the maximum current density is reached in the first cycle (Figure 1C). These results suggest that the increase of catalytic current observed during the initial cycles is linked to a modification, most likely a reorientation, taking place on the adsorbed protein and that O_2_ does not play a role on such process.

The comparison of the maximum current density obtained for each case, demonstrates that activation of CueO upon cycling in the presence of Ar saturated solution gives rise to slightly higher O_2_ reduction current density. Although the maximum current density depends on the activation protocol, the O_2_ reduction onset potential remains similar (between 0.3 and 0.35 V) for the three different experimental situations.

**Figure 2:**
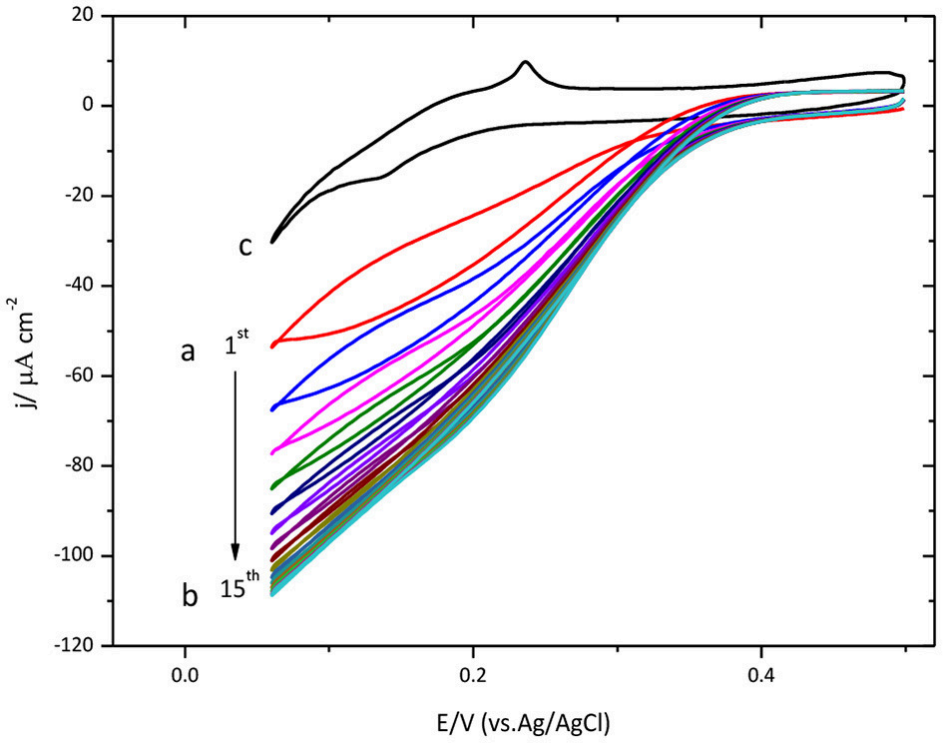
Cyclic voltammogram for CueO immobilized on bare Au(111) in an O_2_ saturated PBS, pH 6.5. (a) Red line: initial cycle. (b) 15th cycle (maximum current density for the ORR). (c) Au(111) blank in O_2_ atmosphere (black line). Scan rate: 20 mVs^−1^.

Protein immobilization was also done on Au(111) surface. The results are shown in Figure 2. Similar to the results described above for glassy carbon, this figure also shows an increase of the catalytic current density during the initial cycles.

The maximum value is reached on the 15th cycle. Nevertheless, the following cycles exhibit a small decrease of the density current.

**Figure 3:**
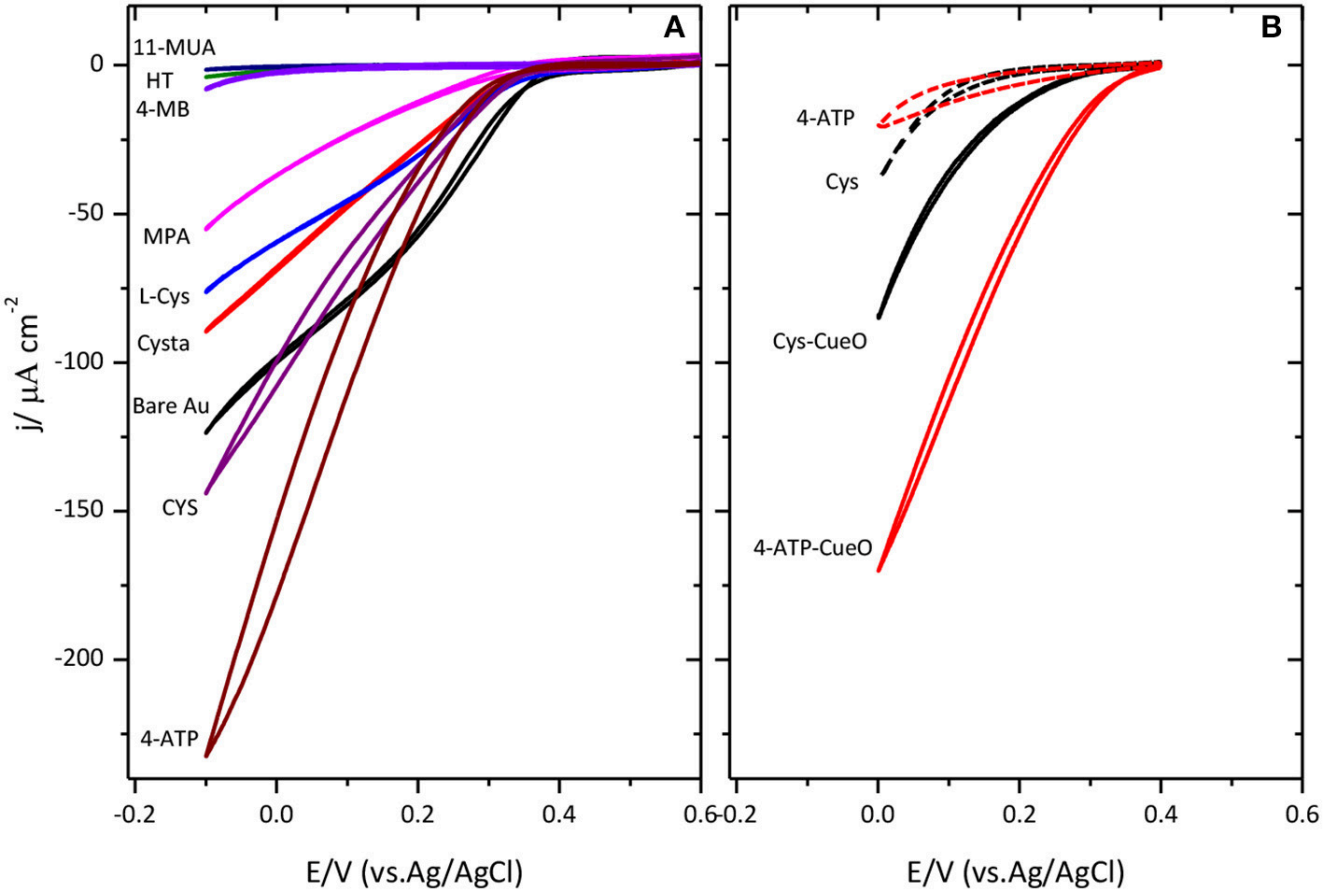
Cyclic voltammograms for CueO immobilized on different SAMs modified Au(111) surfaces (as indicated in **(A)** and poly-Pt electrode modified with CYS and 4-ATP **(B)** in an O_2_ saturated sodium PBS, pH 6.5. Dashed lines in figure **(B)** show the response of the thiol modified Pt surface in absence of protein. Scan rate: 20 mVs^−1^.

Figure 3A collects different cyclic voltammograms for the catalysis of the ORR when CueO is immobilized on Au(111) modified with amino or carboxylic terminal groups self-assembled monolayers. The results reported here agree with previous observations reported in (Climent et al., 2014). In this case, it was concluded that SAMs terminated in amino groups resulted in the maximum catalytic activity while SAMs terminated in carboxylic acid or hydrophobic groups resulted in significantly lower activity. This agrees with the existence of negatively charged residues on the surface of the protein close to the type 1 center (Roberts et al., 2002) that would tend to orientate this area toward the surface when the latter is positively charged, by electrostatic interaction, resulting in a more facile electron transfer between the protein and the electrode surface.

According to these results, platinum modification was also performed using the amino terminated SAMs, namely, 4-ATP and cysteamine, as shown in Figure 3B. Cyclic voltammograms for platinum exhibit the same trend as for gold electrodes. Again, the maximum activity for the ORR is achieved in the case of 4-ATP self-assembled monolayers.

As previously discussed, the initial voltammetric cycles after protein immobilization evolve with time and current increases in each cycle. Another consequence of this current increase is a clearly different shape for the first cycle in comparison with the second and subsequent cycles. This is most likely caused by the reorientation taking place on the protein adlayer during the potential scan. At this point, it is still unclear the role of the potential on such reorganization. To clarify this effect, the following experiments were performed.

**Figure 4:**
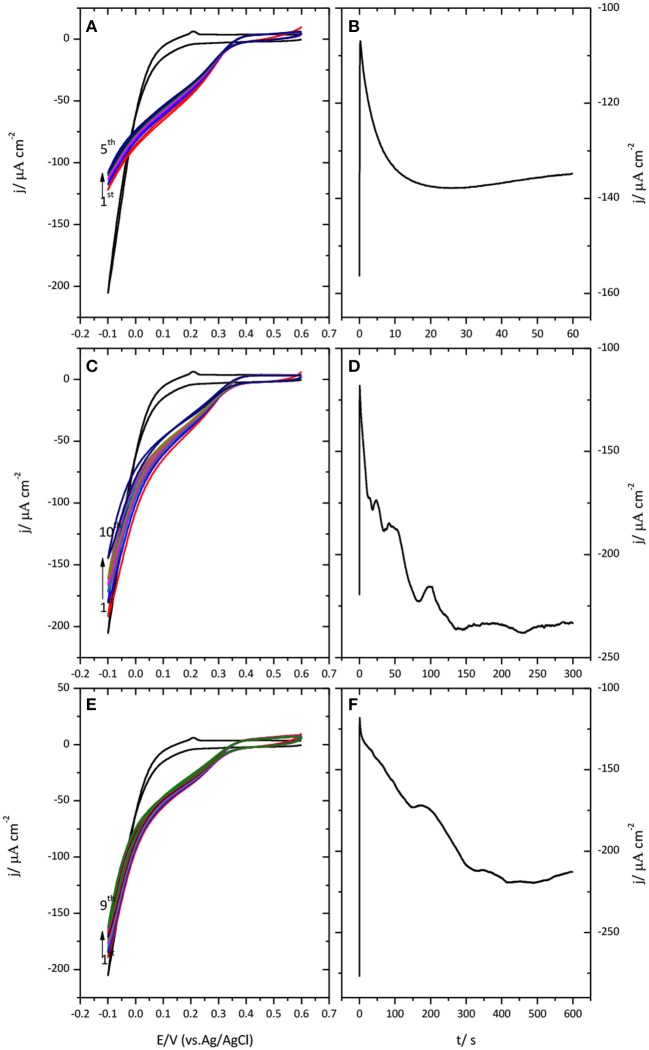
Cyclic voltammogram for the catalysis of the ORR by immobilized CueO on Au(111) after protein submission to −0.1 V during 1 min **(A)**, 5 min **(C)**, and 10 min **(E)**. Monitoring of the CueO current density with time while protein submission to −0.1 V during different periods of time: 1 min **(B)**, 5 min **(D)** and 10 min **(F)**. Scan rate: 20 mVs^−1^.

Figure 4 shows the results obtained when a potential of −0.1 V was applied to the Au(111)-CueO electrode during 1 (A), 5 (B) and 10 (D) minutes, respectively.

Chronoamperometric measurements performed during this polarization time reveal a continuous increase of current that can extend in some cases up to 5 min. Such an increase is followed by a plateau or a slight decrease of current. This evolution suggests the existence of a potential induced reorientation of the protein favored by the application of negative potential values. Subsequent potential cycling shows that polarization of immobilized CueO at this cathodic potential, even for a short period of time of 1 min, is enough to generate a well-defined narrow curve with the highest current density value for the catalysis of the ORR.

The effect of the initial polarization potential was also investigated. For this, prior to the initial potential sweep, the potential was held during 10 min at different values ranging from the open circuit potential (OCP) to −0.1 V. When the initial polarization is done at the OCP, the first voltammetric cycle is wide and undefined and 3 cycles are needed to reach the maximum oxygen reduction current density. After applying a potential of 0.35 V, the first cycle is still wide and undefined; however, only one cycle is enough to achieve the maximum oxygen reduction current density. Meanwhile, when the protein modified electrode is initially polarized at −0.1 V the maximum current density is attained already in the first cycle, as mentioned previously, generating from the beginning a narrow and a well-defined curve. The results of this experiment are summarized in supporting information (Figure S2), where the data for the oxygen reduction catalytic current density for the first four cycles is collected as a function of the initial polarization potential.

An analysis of this result demonstrates that holding low potential values leads to the highest oxygen reduction current density. The more negative the potential is, the lower numbers of cycles are needed to reach the highest oxygen reduction current density and the curves obtained are better defined. After maximum current density is attained, current density decreases in subsequent cycles due to protein desorption or denaturation.

### Immobilization time dependence

As described previously, immobilization of the protein is achieved simply by the immersion of the electrode in the protein solution. However, reported immobilization times span a broad range of values that can go from several minutes (Weigel et al., 2007; Ramírez et al., 2008; Tominaga et al., 2008; Dos Santos et al., 2010; Climent et al., 2012) to many hours (Shleev et al., 2005a; Nogala et al., 2010). For this reason, the influence of this parameter on the immobilization of CueO on gold electrodes was tested. For this, each immobilization time was repeated at least 8 different times to be able to statistically discriminate between the intrinsic irreproducibility of the immobilization process and the true effect of the studied parameter.

Protein immobilization times of 1, 5, 10, 20, and 40 min, respectively, reveal that short times give rise to current density values situated between 60 and 125 μA cm^−2^. No significant differences are obtained in the subsequent catalytic activity in this range of immobilization times. However, immobilization times of 2 h or longer produce significantly lower current density values (below 20 μA cm^−2^) for the ORR. A comparison between long and short immobilization times indicates that long times result in maximum current density values about 30 percent of the results obtained for short times. A graphic comparison of the maximum current achieved on the different experiments is shown in supporting information, Figure S3.

### CueO immobilization by covalent bond

In order to increase the stability and the number of protein molecules immobilized on the electrode surface, CueO covalent immobilization on modified gold electrodes was performed. Peptide bonds can be formed between the protein and the adequate functional group on the surface of the electrode to increase the interaction strength of the immobilization. To achieve this, carboxylic groups on the surface of the electrode can react with amine groups on the protein, mainly from lysine residues. Conversely, amine groups on the surface of the electrode can react with carboxylate groups on the protein. This strategy usually implies the use of a reagent to activate the carboxylate groups by a sterification reaction that leads to the formation of a good leaving group. With this idea, activation of carboxylic functional groups on an MPA SAM was first carried out using three different mixtures of NHS/EDC and a common activation time of 1 h. Then, the electrode was immersed in the corresponding protein sample to achieve the formation of amide bonds between amine groups on the surface of the protein and the activated carboxylate groups on the SAM. The obtained results clearly demonstrate that catalytic current density values obtained for protein covalently bonded to the modified electrode surface are higher than the corresponding catalytic values recorded for protein physically adsorbed on the modified electrode surface.

**Figure 5:**
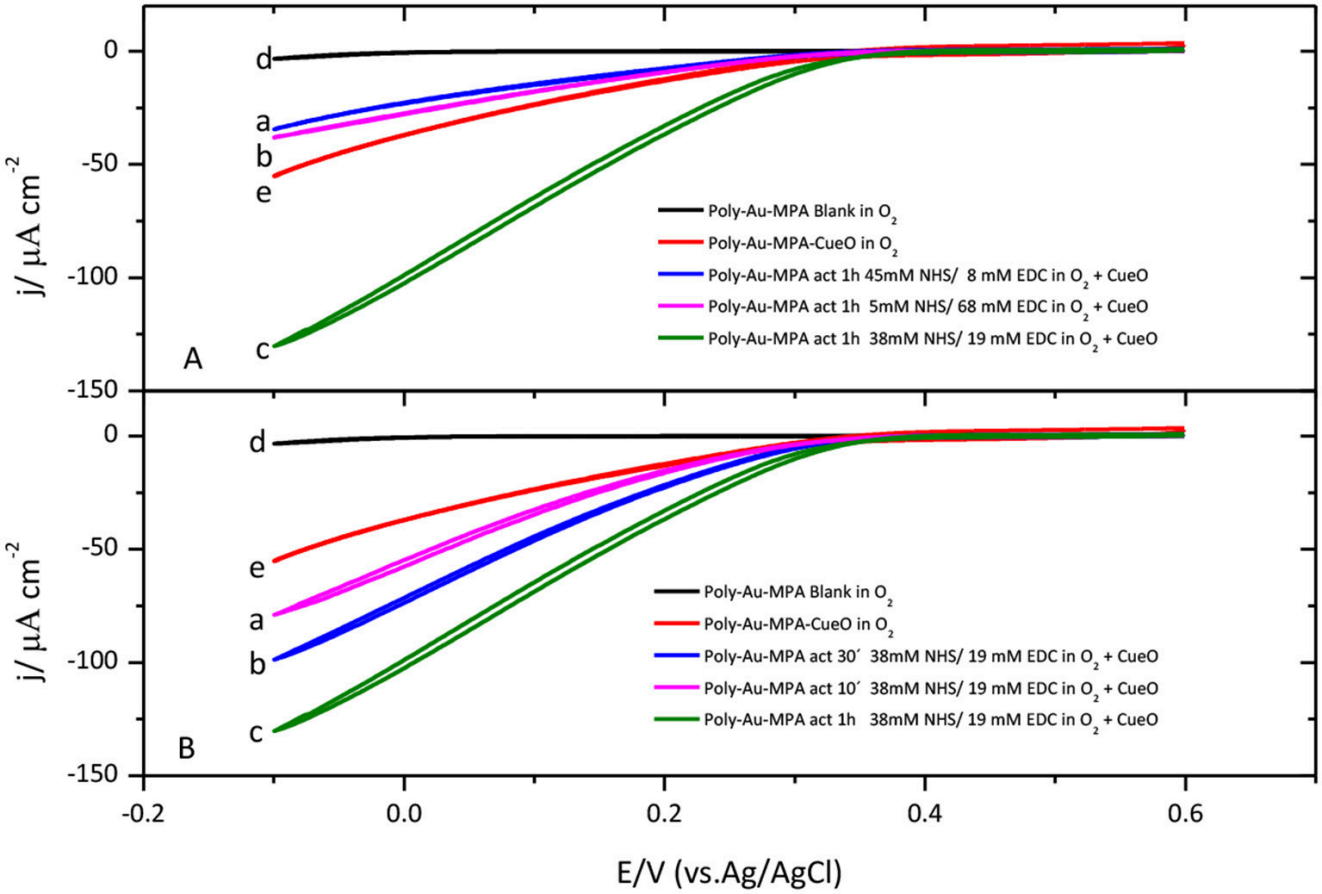
**(A)** Cyclic voltammograms for CueO immobilized (1 h) on MPA SAM after activation in the presence of different NHS/EDC mixture solutions: (a) blue line (45 mM NHS/8 mM de EDC), (b) pink line (5 mM NHS/68 mM de EDC), (c) green line (38 mM NHS/19 mM de EDC). (d)Black line: Poly-Au-MPA blank. (e) Red line: Poly-Au-MPA-CueO. **(B)** Cyclic voltammograms for CueO immobilized 10 min (a, pink line), 30 min (b, blue line) or 1 h (c, green line) on MPA SAM after activation in the presence of a 38 mM NHS/19 mM EDC mixture. d) Black line: Poly-Au-MPA blank. e) Red line: Poly-Au-MPA-CueO. O_2_ saturated PBS. Scan rate: 20 mVs^−1^.

Different activation times and different concentrations of the reagents were attempted to optimize the immobilization process. The highest catalytic current for the O_2_ reduction by CueO is obtained by activation of the SAM carboxylic groups during 1 h in a mixture of NHS 38 mM and EDC 19 mM (Figure 5A). On the other hand, Figure 5B shows that, under these experimental conditions, the use of activation times smaller than 1 h lead to lower O_2_ catalytic current densities while activation times higher than 1 h do not improve the results in terms of catalytic current density.

As previously mentioned, an alternative approach to achieve the covalent bonding of the protein is the activation of the carboxylate groups on the surface of the protein to form the amide bond with an amine terminated SAM on the surface of the electrode. For this, a polycrystalline gold electrode modified with cysteamine or 4-aminothiophenol was immersed into the activated protein solution leading to the formation of amide bonds between the protein and the electrode surface. In this case, analysis of the catalytic current density values (results are shown in Figure S4) indicates that covalent immobilization through the protein carboxylic groups activation does not improve the protein performance with respect to the catalytic behavior of the physically adsorbed protein. The different catalytic activity achieved through both covalent immobilization approaches must reflect the different distribution of amine and carboxylate groups on the surface of the protein.

#### pH effect

**Figure 6:**
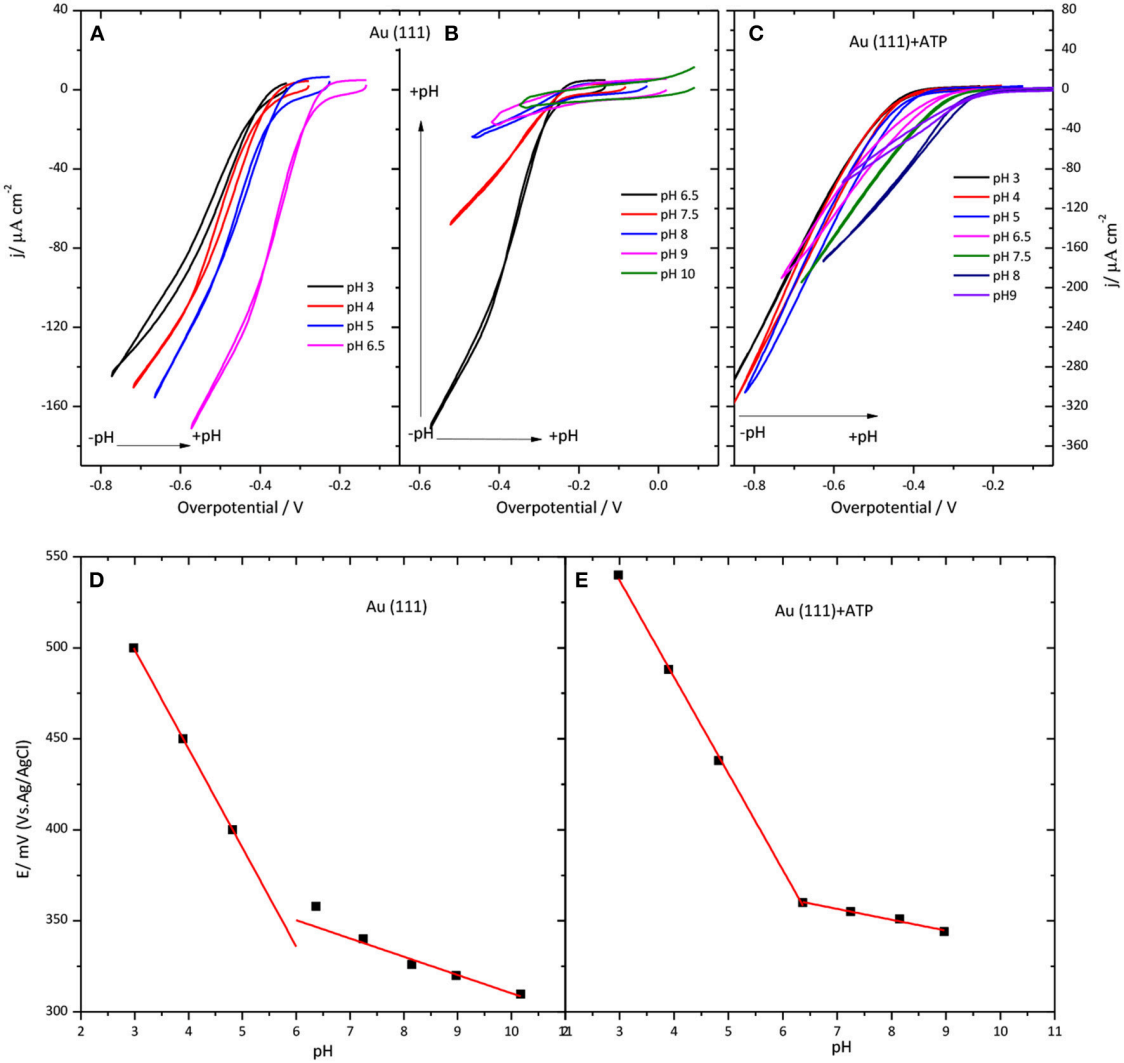
Behavior of CueO on Au(111) **(A,B,D)** and Au(111) modified with 4-ATP **(C,E)** in an O_2_ solution at different pHs values between 3 and 10. **(A–C)** Collect data for the ORR current density against the overpotential of the O_2_ reduction. Scan rate: 20 mVs^−1^. **(D,E)** show the pH dependence of the $E_{\text{T}1-}^{{}^{\circ}}$CueO on Au(111) **(D)** and Au(111) modified with 4-ATP **(E)**. Black solid squares: Experimental values for the $E_{\text{T}1\approx }^{{}^{\circ}}$ $E_{\text{onset}}^{{}^{\circ}}$. Red line: linear fit to the experimental data.

Another investigated parameter was the influence of the pH of the solution on the CueO catalytic behavior. Figure 6 illustrates the results obtained for immobilized CueO on bare Au(111) and 4-ATP modified Au(111) electrodes.

Because the equilibrium potential for the ORR also shifts with the pH, the voltammetric current in Figure 6 is plotted against the overpotential to compensate for this effect. (Figure S5 in Supporting Information shows the current density values against the potential for modified gold electrodes in the pH region between 3 and 9). The ORR onset changes to negative overpotentials with increasing pH. This means that the shift of the curve to lower potentials with increasing the pH is slower than the equilibrium potential. Therefore, when plotted against the overpotential it results in a net positive shift. On the other hand, the increase of the pH of the solution leads to a decrease of the current densities for both unmodified Au(111) and 4-ATP modified Au(111) systems.

The change of the onset potential as a function of the solution pH for both modified and unmodified gold electrodes is reported in Figures 6D,E. According to this figure, the onset potential of both electrodes moves to lower values with an average of 54 mV per pH unit when pH increases from 3.0 to 7.0, in accordance with previous studies of this or similar enzymes (Otsuka et al., 2007; Weigel et al., 2007; Tsujimura et al., 2008; Miura et al., 2009; Clot et al., 2012; Filip and Tkac, 2014). However, the diminution of the onset potential becomes < 54 mV per pH unit for pH values higher than 6.0. In fact, in the case of 4-ATP modified gold electrodes, the onset potential remains nearly constant for pH values higher than 6.5.

### Effect of oxygen concentration

**Figure 7:**
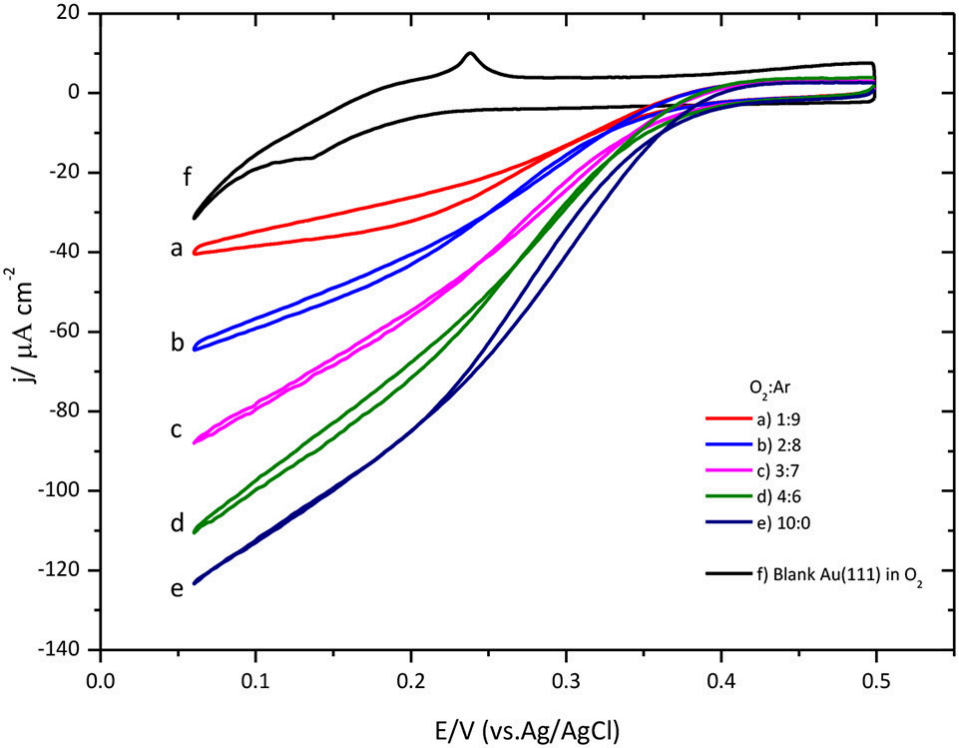
O_2_ concentration effect over CueO cyclic voltammogram curves for protein immobilized on bare Au(111), pH 6.5. Different O_2_: Ar proportions are indicated in the graph. Scan rate: 20 mVs^−1^.

The effect of the O_2_ concentration on the ORR catalyzed by CueO is described in Figure 7.

A close look at the data indicates that low substrate concentrations generate well-defined sigmoidal curves reaching a limiting current density at low potentials. Conversely, using high substrate concentrations, it is impossible to determine the limiting current density value due to the absence of a defined plateau in the cyclic voltammogram. Moreover, the increase of the O_2_ concentration in solution causes a shift of the ORR onset potential to negative values (Figure S6). These findings have been taken before as indication of a possible change in the limiting step for the O_2_ reduction mechanism by the protein (Dos Santos et al., 2010; Climent et al., 2012) corroborating the notion that the limiting step at low O_2_ concentration is the intramolecular electron transfer, instead of the electron transfer between the electrode and the protein.

The Michaelis-Menten equation

(1) $$j=\frac{j_{max}[O_{2}]}{{K^{\prime }}_{M}+[O_{2}]}$$

has been used to determine the characteristic kinetic parameters of the immobilized CueO protein. ${K^{\prime }}_{M}$ and j_max_ for the ORR catalyzed by the enzyme were calculated following the procedure described in references (Welinder et al., 2007; Dos Santos et al., 2010; Climent et al., 2012). ${K^{\prime }}_{M}$ and *j*_max_ are the apparent Michaelis-Menten constant and the maximum current density value, respectively.

**Figure 8:**
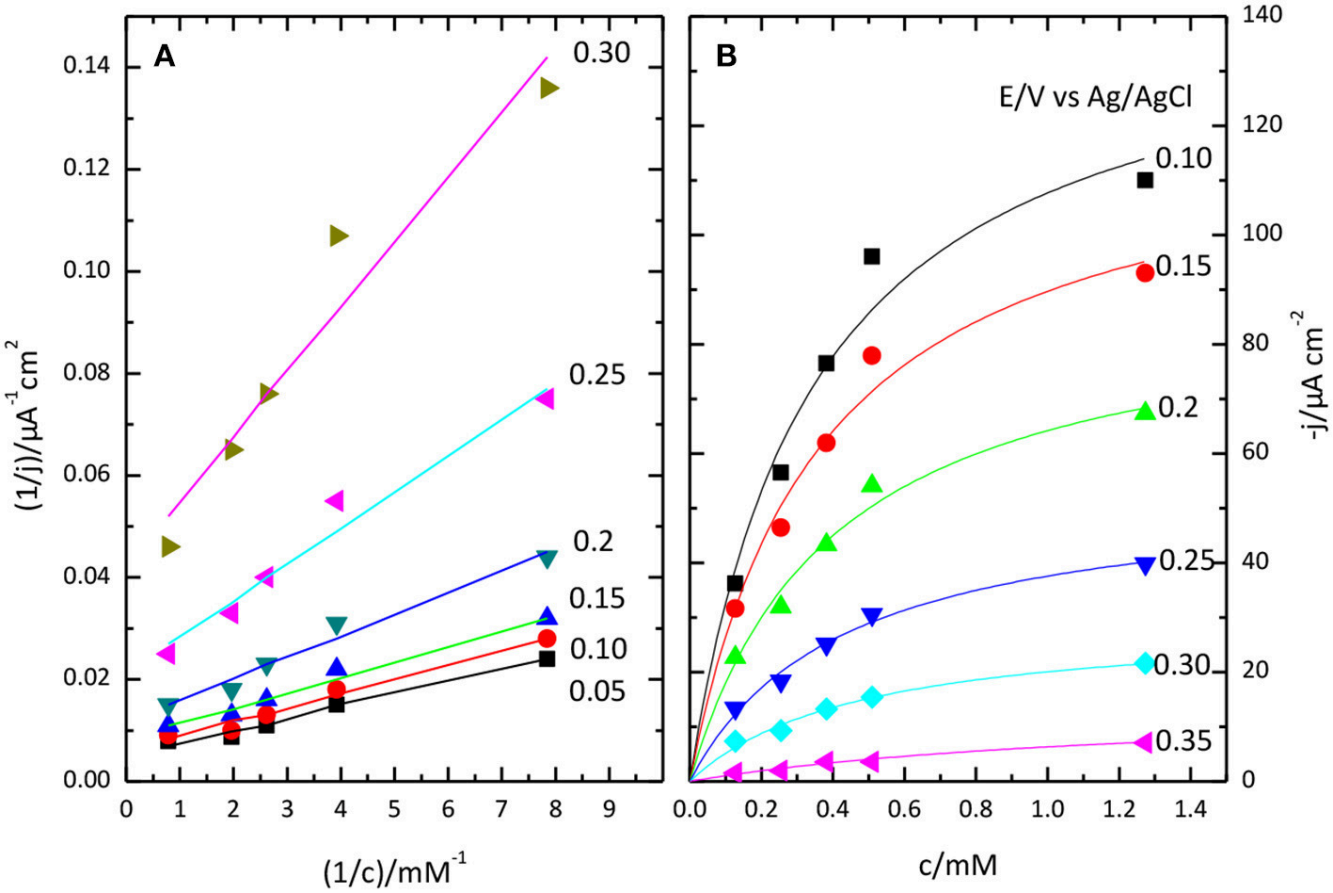
Lineweaver-Burk plot **(A)** and Michaelis-Menten plot **(B)** showing the effect of O_2_ concentration on catalytic current sodium PBS, pH 6.5.

**Figure 9:**
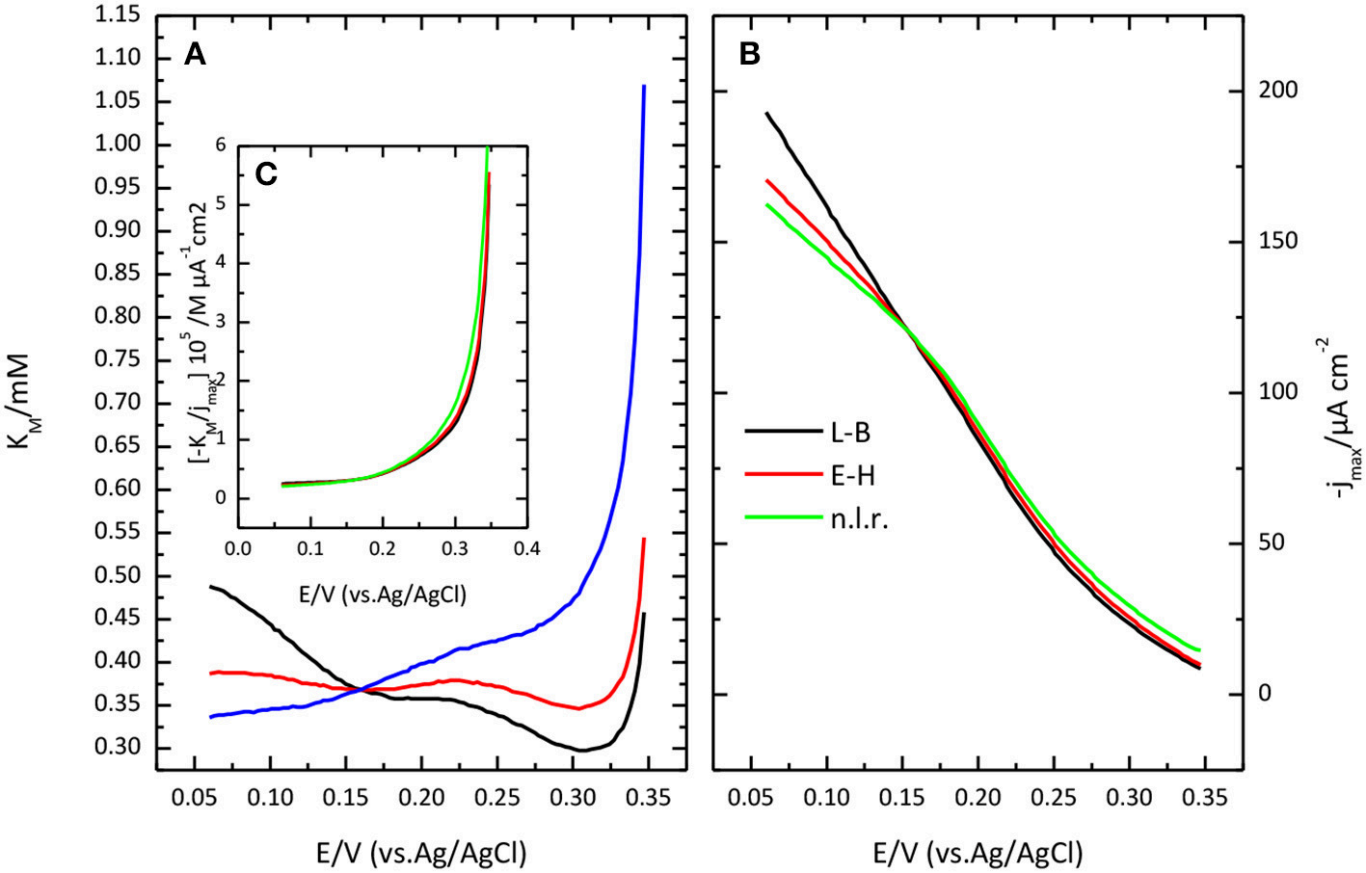
Potential dependence of Michaelis Menten parameters: *K'*_M_ **(A)** and *j*_max_ **(B)** as obtained from Lineweaver-Burk plot (black lines), from Eadie-Hofstee (red lines) or from a nonlinear regression of the data (red lines). **(C)** plot of the ratio between *K'*_M_ and *j*_max_, as a function of potential.

Lineweaver-Burk plot (A) and the fitting of the Michaelis-Menten equation to the experimental data (B) are shown in Figure 8. The O_2_ concentration was determined from Henry's law using 1.27 10^−3^ M as solubility data for a temperature of 298 K. The apparent resulting values for ${K^{\prime }}_{M}$ and *j*_max._ as a function of potential are given in Figure 9. In general, the nonlinear regression method to extract catalytic parameters is preferred since it gives equal weight to the different data points, while Lineweaver-Burk plot tends to give higher weight to the points at lower concentration. An alternative approach is the so called Eadie-Hofstee plot, where *j* is plotted vs. *j*/[O_2_] (Dowd and Riggs, 1965). The result of this analysis is also included in Figure 9. The three procedures provide similar values of ${K^{\prime }}_{M}$ and *j*_max._ The results indicate a marked dependence of ${K^{\prime }}_{M}$ and *j*_*max*_ with the potential. ${K^{\prime }}_{M}$ tends to a value of ca. 0.35 mM at low potentials. This result is similar to that reported for related enzymes such as Bilirubin Oxidase (BOx) (Dos Santos et al., 2010) and the laccase from *Streptomyces coelicolor* (Climent et al., 2012).

The potential dependence of those parameters have been previously analyzed in the framework of a simplified kinetic model involving an electron transfer step followed by a catalytic step (Climent et al., 2012).

**Scheme 1:**
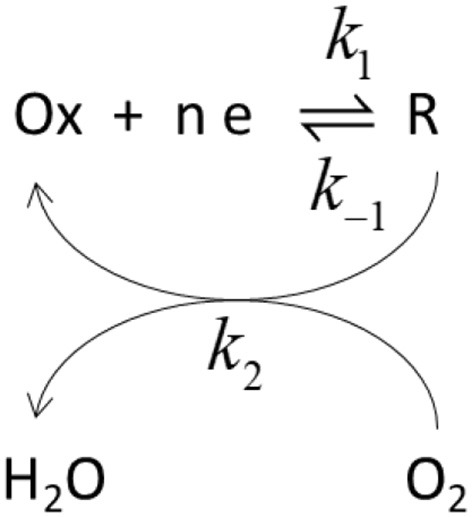
Schematic representation of the catalytic cycle. k_1_ and k−1 represent rate constants for the electron transfer while k_2_ is the rate constant for the catalytic step.

In this Scheme 1, Ox and R represent the oxidized and reduced states of type 2/3 copper atoms. The conversion between oxidized and reduced states in the real protein involves the transfer of electrons from the type 1 copper center. If such intramolecular electron transfer is fast, then *k*_1_ and *k*–_1_ can be considered as rate constant for the electron transfer from the metal. The true parameters of the Michaelis-Menten kinetic model affect *k*_2_, according to (Welinder et al., 2007):

$$\begin{array}{l} k_{2}=\frac{k_{2m}}{K_{M}+\left[O_{2}\right]} \end{array}$$

In this framework, the apparent dependence of the parameters ${K^{\prime }}_{M}$ and *j*_max_ comes from the effect of the electrode potential on *k*_1_ and *k*–_1_. The ratio of ${K^{\prime }}_{M}$ over *j*_max_, i.e., the slope of the lines in Lineweaver-Burk plot, tends to a constant value at high overpotential, when *k*_1_ >> *k*–_1_, equal to the ratio of the true, potential independent values of *K*_M_ and *F*Γ*k*_2*m*__._where Γ is the coverage of active centers. Such a ratio is also plotted in Figure 9C. The limiting value at low potential of this ratio is ca. 2.5 10^−6^ M μA^−1^ cm^2^, with a value for the potential independent *K*_M_ around 0.4 mM.

#### Thermal stability

**Figure 10:**
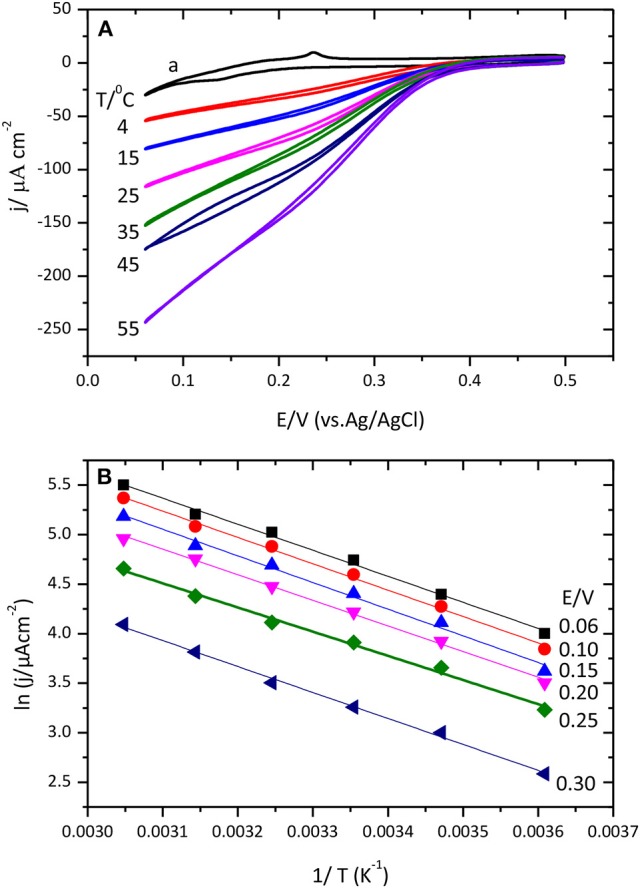
**(A)** Cyclic voltammograms for the catalytic O_2_ reduction by CueO immobilized on unmodified Au(111) electrode in an O_2_ saturated PBS, pH 6.5, using different temperatures between 4 and 55 °C. Curve (a) corresponds to the blank Au(111) at 25°C. **(B)** Data representation of ln j vs. 1/T (Linear Arrhenius plot).

Figure 10A shows cyclic voltammograms for the catalysis of the O_2_ reduction reaction by CueO protein at different temperatures.

Inspection of the voltammetric curves demonstrates a clear dependence of the ORR with temperature. Temperature increase leads to higher current density values and shifts the O_2_ reduction onset potential to positive values facilitating the beginning of the reduction reaction. The evolution of the cyclic voltammogram during the initial potential cycles after protein immobilization is also interesting. Cyclic voltammograms collected at 4°C need around 30 cycles to reach the maximum value; while at 55°C just two cycles are needed to attain the maximum current density value. From this second cycle, the current density either stays nearly constant or starts to decrease slightly (Figure S7). This clearly points toward the existence of a reorganization process that leads to a more efficient conformation but that requires to overcome a relatively high activation energy to break the various interaction points between the protein and the surface. Another noteworthy aspect is the variation of the shape of the first voltammetric cycle with the temperature: high temperatures generate wider and less defined curves for the initial voltammetric cycle than low temperatures. This is most likely reflecting the changes in the protein conformation during the voltammetric cycle resulting in a distorted voltammetric shape. At low temperatures, however, the reorganization of the protein and the voltammetric cycle take place in different time domains. Furthermore, the increase of the temperature produces an increase of the value of the ORR current measured in the first cycle.

**Figure 11:**
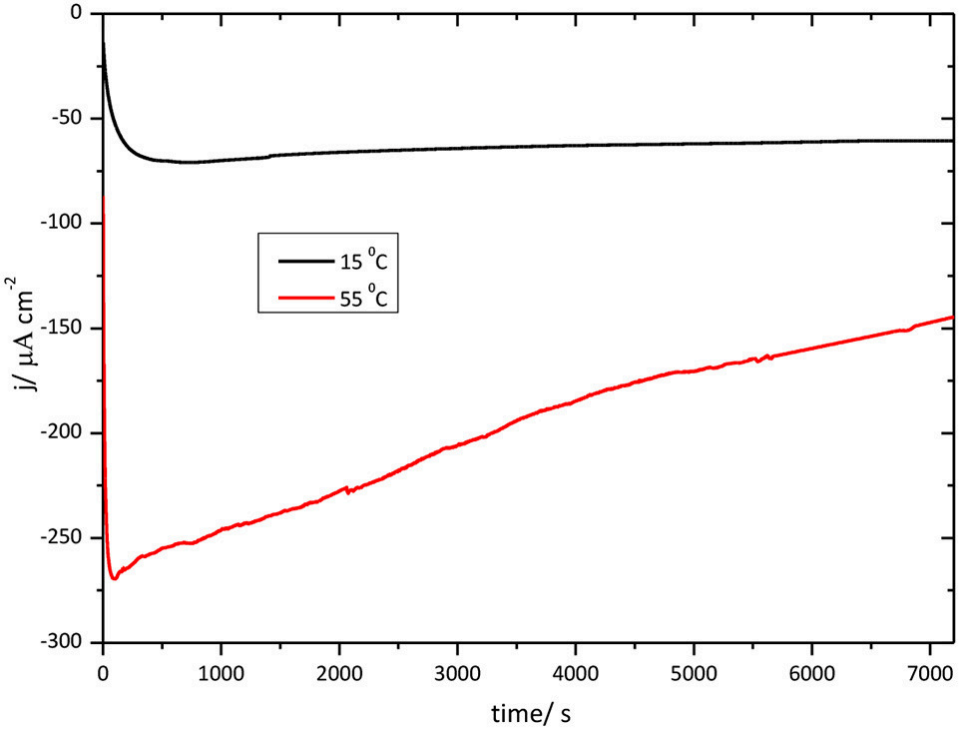
Chronoamperometry for the catalytic O_2_ reduction by CueO immobilized on unmodified Au(111) electrodes in an O_2_ saturated PBS, pH 6.5, Black line: chrono at 15°C. Red line: chrono at 55°C.

Figure 11 shows chronoamperometric results for the O_2_ reduction reaction catalysis by immobilized CueO on bare Au(111) at different temperatures.

In order to evaluate the thermal stability of CueO with time, the current density evolution was followed during 2 h for each temperature. The initial portion of the curve shows the effect of the temperature on the activation process just mentioned above. O_2_ reduction reaches the maximum current density for the measurement at 55°C, however, after 2 h working at this high temperature, the current density decreases reaching nearly 60% of the initial value. On the other hand, at 15°C, the O_2_ reduction current density catalyzed by CueO keeps nearly constant throughout the 2 h of operation.

According to the Arrhenius equation (2) the apparent energy of activation for the ORR can be calculated by plotting ln *j* (where *j* is the current density magnitude registered at different potential values and for different temperatures) as a function of *T*^−1^ (being *T* the experimental temperature)

(2) $$\begin{array}{l} K=Ae^{\frac{-Ea}{RT}} \end{array}$$

An experimental apparent activation energy value of 21.6 kJ mol^−1^ has been calculated from the slopes of the linear fittings obtained for the experimental data in Figure 10B. The magnitude of this activation energy is in agreement with result of previous studies with the related enzyme BOx or less related hydrogenases (Dos Santos et al., 2010; Hexter et al., 2012). Results on Figure 10B show similar slope variation for data of lnj Vs T^−1^ (at different temperatures), which means that CueO apparent activation energy values are not potential dependent.

#### Inhibitors

**Figure 12:**
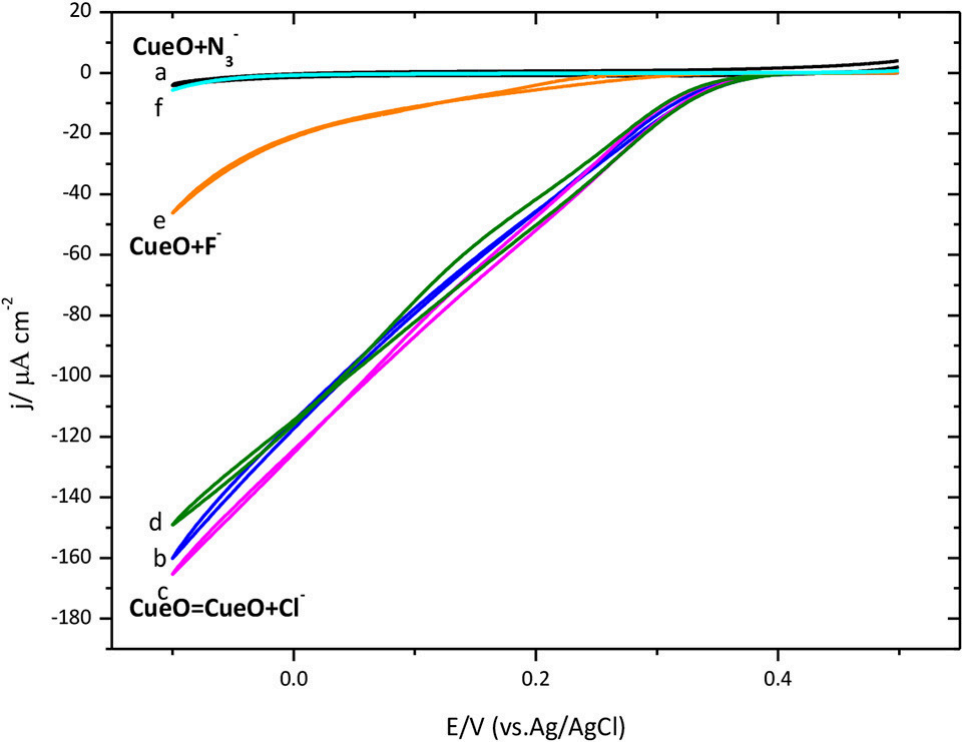
Cyclic voltammograms for the catalytic O_2_ reduction by CueO immobilized on a poly-Au-CYS modified electrode in an O_2_ saturated PBS pH 6.5, using different inhibitors (as indicated). (a) Poly-Au-CYS blank (black line, 20 mV/s), (b) poly-Au-CYS-CueO (blue line 2 mVs^−1^), (c) poly-Au-CYS-CueO-100 mM NaCl (pink line), (d) poly-Au-CYS-CueO-400 mM NaCl (green line), (e) poly-Au-CYS-CueO-100 mM NaF (orange line), (f) poly-Au-CYS-CueO-100 mM NaN_3_ (cyan line).

In this part we describe the effect on the activity of immobilized CueO of some compounds, normally recognized in the literature as inhibitors of the catalytic process of laccases. Figure 12 shows the activity of the enzyme for O_2_ reduction in the presence of chloride, fluoride and azide.

As discussed previously, an increase of the current during the initial cycles suggests a reorientation of the immobilized protein toward a configuration where the electron transfer is favored. In Figure 12, the maximum current after the initial cycles is shown. Then, the inhibitor was added into the electrochemical cell and a new voltammetric cycle was recorded to test the inhibitory effect.

Data indicates that F^−^ and $N_{3}^{-}$ behave as inhibitors of the catalytic ORR suppressing the activity of CueO protein. However, the reduction current density in the presence of 100 and 400 mM Cl^−^ remains unchanged suggesting that Cl^−^ does not affect the CueO catalytic activity for the ORR.

**Figure 13:**
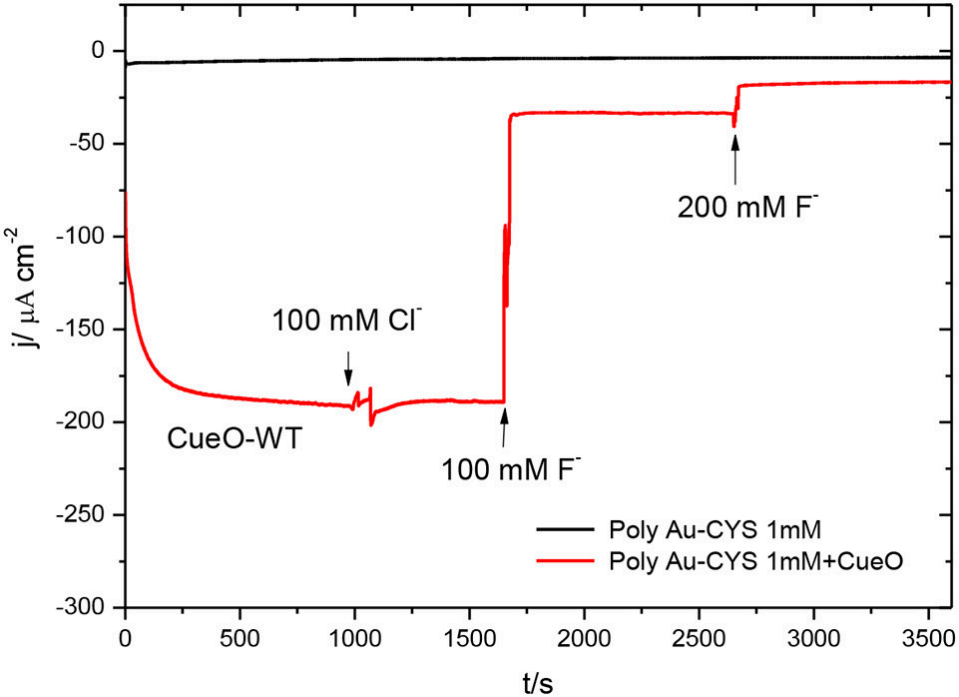
Chronoamperometric measurements in an O_2_ saturated PBS: poly-Au-CYS modified electrode (black line), poly-Au-CYS-CueO modified electrode in absence of inhibitors (red line, until *t* = 1,000 s), in the presence of 100 mM Cl^−^ concentration (red line, until *t* = 1,650 s), 100 mM F^−^ concentration (red line, until 2,650 s) and 200 mM F^−^ concentration (red line, until 3,580 s).

Figure 13 shows the result of chronoamperometric experiments measured during 1 h for CueO immobilized on a poly-Au-CYS electrode in the absence and in the presence of Cl^−^ and F^−^.

The data in this figure reveal that addition of Cl^−^ does not affect the ORR current density. On the other hand, the current density decreases significantly after the addition of fluoride up to a concentration of 100 mM. Moreover, the addition of an even higher concentration of F^−^ produces a further decrease of the current density, reaching values close to those obtained in the absence of protein (black line).

Finally, reversibility of the inhibition by F^−^ and $N_{3}^{-}$ was also evaluated. To carry out this experiment two electrochemical cells were employed. The first cell was used to record the cyclic voltammogram for the CueO modified electrode in an oxygen saturated sodium phosphate buffer solution in the absence of inhibitors. The evolution of the current density with the potential cycling is registered until the maximum constant value is reached. Then, the electrode was transferred into a second cell, which contained 100 mM F^−^ or $N_{3}^{-}$ and a new potential cycle was registered. Data recorded in this second cell reflect the diminution of the current density due to the inhibitory effect over the catalytic CueO activity. After that, the electrode was again transferred back into the first cell where no inhibitors were present. The reversibility of the inhibition process is complete in the case of F^−^ and virtually all the initial activity is recovered, while in the case of $N_{3}^{-}$ the recovery is only partial. These results are shown in Figure S8.

#### CueO concentration

We have also investigated the effect of CueO concentration in the solution used for the immobilization of the protein. For this, we performed the immobilization using aliquots with different protein concentration, but the same immobilization time for each sample (5 min). The available evidence seems to point out that there is no clear trend that implies an increase of the current density with an increase or decrease of CueO concentration, Figure S9 in Supporting Information.

## Discussion

The results described above demonstrate that immobilized CueO protein on unmodified glassy carbon or (bare or thiol modified) gold and platinum electrodes catalyzes the electrochemical ORR. The maximum current obtained in the present case is the same order of magnitude as that previously reported with related enzymes using a similar immobilization procedure. For instance, around 300 μA cm^−2^ were reported in (Dos Santos et al., 2010) using BOx, although this figure could be increased to ca. 1,000 μA cm^−2^ after modification of the surface with diazonium chemistry. Using BOx on a gold nanoparticle gold surface, values of current as high as 5,000 μA cm^−2^ were reported in (Murata et al., 2009). Much lower values, around 30 μA cm^−2^ are reported for BOx on gold in (Ramírez et al., 2008). Finally, the maximum current achieved with several laccases reported in (Shleev et al., 2005a) is also in the range 40–200 μA cm^−2^.

One particular behavior observed for the present system is that, for all the studied substrates, the O_2_ reduction voltammetric currents significantly increase during the initial cycles or initial polarization time. The detailed reason for this growth is still unclear. We hypothesized that the current density increase indicates a protein reorientation toward a configuration that favors DET with the substrate. Chronoamperometric experiments have revealed a clear role of the electrode potential in this reorganization, since this process does not occur for the oxidized form of the protein or under open circuit conditions. Also, the reorganization is faster if low potentials are applied. Since the region close to the type 1 copper center is rich in sulfur containing aminoacids, one tentative explanation would be the reductive formation of thiol bonds with the surface. Higher temperatures also favor this reorganization process either because they favor the rupture of bonds with the surface or because they increase the mobility of the protein.

While non-turnover signal (in the absence of O_2_) for immobilized protein is not observed on gold or platinum substrates, the appearance of a couple of redox peaks after activation of the immobilized CueO on glassy carbon by cycling the potential in anaerobic conditions (Ar saturated solution), suggests that the direct electron transfer (DET) also takes place under non catalytic conditions. Similarity of the potential value of these redox peaks with the reported data for the ${\text{E}}_{\text{T1}}^{\text{o}}$(Tsujimura et al., 2008; Miura et al., 2009) provides support to assign the type 1 copper site as the electron entrance when protein is immobilized on unmodified glassy carbon surfaces in accordance to previous spectroscopic measurements on gold (Climent et al., 2012). The absence of non-catalytic signal in the case of gold or platinum substrates might just be related with a much lower surface coverage in those cases.

Stability of the current is higher on glassy carbon or the thiol modified gold surface. On the other hand, the existence of an inactivation process after the maximum current is attained on the bare gold surface may be due either to the protein denaturation upon potential cycling or the diffusion of protein into the solution.

Experiments performed using Au(111) or poly-Pt modified with different self-assembled monolayers were executed in order to design chemical systems that simulate natural environments in which enzymes operate and serve as a link between the electrode and the biomolecule. Self-assembled monolayers interact with the protein depending on its hydrophobic/ hydrophilic properties, allowing protein immobilization without damage of its tertiary structure. The catalysis of the ORR by CueO immobilized on Pt and Au modified with 4-ATP produces the maximum O_2_ reduction current density (Climent et al., 2014). This result supports that CueO can be immobilized on gold using amino terminal self-assembled monolayers that interact with the aspartic or glutamic acid residues close to the protein active sites. Current research for platinum appears to validate such a view.

Anchoring methods based on the formation of covalent bonds between the electrode and the enzymes are efficient procedures to establish strong protein-substrate interaction (Vaz-Dominguez et al., 2012). The formation of amide bonds (either between the carboxylic groups of the activated SAM and the amine groups of protein aminoacids or between the protein activated carboxylic groups and the amine groups of the SAM; Willner and Katz, 2000; Klis et al., 2007) results in strong protein-surface interaction. While physical protein immobilization by just immersing the electrode into the protein solution leads to a weak adsorption on the electrode surface, covalent immobilization ensures better protein stability that it is reflected in higher currents, most likely due to higher values of coverage or better orientation of the immobilized protein, while current remains stable for longer times. The most important drawback of this kind of covalent immobilization method is the possible perturbance of the protein active site during the activation process although in the case reported here this problem seems to be absent since the catalytic protein activity remains unchanged.

The next discussion regards the effect of the pH on the catalytic activity of immobilized CueO over the pH range 3–9. Judging from the results of current density against the potential (shown in supporting information) it seems that protein bioelectrocatalytic activity is higher in acid than in neutral or basic media, but this is just due to the shift of the equilibrium potential with pH. Opposite to this, the change of the current density with the overpotential (Figure 6) demonstrate that protein catalytic behavior toward the ORR is indeed higher in neutral than in acid media since the ORR starts at lower overpotentials for neutral than for acid pH. Based on the results described above, the existence of DET between CueO protein and the electrode and the entrance of the electrons from the electrode through the type1 site allow to follow the evolution of the type 1 copper site formal potential ($E_{\text{T}1}^{{}^{\circ}}$) with the pH. Experimental $E_{\text{T}1}^{{}^{\circ}}$ change with the solution pH was analyzed by following the onset potential where current density starts to increase. Such variation suggests that proton transfer plays an important role in the monoelectronic redox reaction associated to the T1 copper site. The $E_{\text{T}1}^{{}^{\circ}}$ variation with the pH is less intense when CueO works in basic than in acid media and $E_{\text{T}1}^{{}^{\circ}}$ becomes independent of the pH at high pH values.

Experiments at different temperatures highlight a major protein catalytic activity when working at high temperatures (since the highest O_2_ reduction current density is reached at 55°C), however protein deactivation is also faster as the temperature is higher. However, working at low temperature conditions, CueO generates lower O_2_ reduction current densities, although in this condition the protein can operate without current decay for longer times than at high temperatures.

Finally, the effect of the presence of inhibitors that actuate at different stages of the active cycle is investigated. In the present work, the catalysis of the ORR in the presence of some inhibitors such as F^−^, Cl^−^, and $N_{3}^{-}$ was evaluated. The data show that in the presence of fluoride and azide there is an important decrease of the O_2_ reduction current density. Fluoride is described as a non-competitive inhibitor of the direct electron transfer for *Trametes hirsuta* or *Polyporus versicolor* (Naqui and Varfolomeev, 1980; Pita et al., 2006; Vaz-Dominguez et al., 2008; Salaj-Kosla et al., 2013). Consequently, both F^−^ and $N_{3}^{-}$can be described as inhibitors of the DET for CueO. On the other hand Cl^−^ is portrayed as a competitive inhibitor (Vaz-Dominguez et al., 2008; Salaj-Kosla et al., 2013), which works by blocking the access to the type 1 site and inhibiting the mediated catalytic activity of mediators such as ABTS or 2,6-DMP. Another interesting aspect regarding the current cyclic voltammetric data for the catalysis of the ORR by CueO in a solution containing chlorides is that the current density remains constant even in an electrolyte buffer solution containing chlorides. These findings indicate that chlorides neither block the access of the electrons from the electrode to the type 1 site nor from the type 1 site to the type 2/3 copper site. Thus, CueO retains it catalytic activity toward the ORR since the direct electron transfer mechanism is not blocked in the presence of Cl^−^ ions. Similar behavior is registered in case of other laccases from *T. hirsuta* (Vaz-Dominguez et al., 2008) or *P. versicolor* (Naqui and Varfolomeev, 1980).

Moreover, in this paper we prove that the inhibition of the CueO catalytic activity is not an irreversible process, since it is possible to recover most of the enzyme catalytic capacity just by cycling the inhibited-protein in a sodium electrolyte phosphate buffer solution free of inhibitors. In fact, data for fluoride and azide indicate a total reversibility in regard to the inhibition process of the ORR catalysis of fluorides and a partial reversibility with respect to the azide effect.

## Conclusions

It has been found that immobilized CueO on different electrode surfaces, such as glassy carbon, gold or platinum unmodified or modified with different self-assembled monolayers, preserves its catalytic activity toward the O_2_ reduction to H_2_O, with typical values of catalytic current ranging between 130 and 200 μA cm^−2^ at 25°C, pH 6.5 and 0.05 V vs. Ag/AgCl. Direct electron transfer bielectrocatalysis is observed in case of immobilized CueO on bare glassy carbon and bare and SAM modified gold and platinum surfaces. We have reported an optimal CueO immobilization time situated between 1 and 40 min while protein immobilization times equal or higher than 2 h generate some inactivated protein films that prevent the electron transfer between the electrode and the enzyme. CueO activity for the ORR is higher in neutral than in acid media. CueO produces low O_2_ reduction current densities at low temperatures but its resistance for the current decay is bigger at low than at high temperatures. Enzyme submission to different potential values implies a protein reorientation process that favors the most active conformation for the connection electrode-protein and the subsequent ORR to H_2_O. Finally, F^−^ and $N_{3}^{-}$ inhibit the CueO catalytic activity toward the ORR, however in the presence of Cl^−^ the protein retains its bioelectrocatalytic properties.

## Author contributions

VC, BM, and JF designed the experiments. BM designed and carried out the protein purification. SC carried out the electrochemical experiments and protein purification. SC and VC performed the initial analysis of results. SC and VC wrote the initial version of the ms. while all authors participated in the discussion of results and edition of the final version of the ms.

### Conflict of interest statement

The authors declare that the research was conducted in the absence of any commercial or financial relationships that could be construed as a potential conflict of interest.
