# Supplementary material for: Comprehensive Study of the Enzymatic Catalysis of the Electrochemical Oxygen Reduction Reaction (ORR) by Immobilized Copper Efflux Oxidase (CueO) From Escherichia coli
Source: Front Chem. 2018 Aug 24;6:358. doi: 10.3389/fchem.2018.00358 (PMC6117412; doi:10.3389/fchem.2018.00358)
Supplement: Supplementary file 1 [file Image_1.pdf]

SUPPORTING INFORMATION FOR:

Comprehensive study of the enzymatic catalysis of the Electrochemical Oxygen Reduction Reaction (ORR) by immobilized Copper efflux oxidase (CueO) from *E.Coli*

Sara Chumillas, Beatriz Maestro, Juan Feliu and Victor Climent

(1) *Institute of Electrochemistry, University of Alicante, Alicante, Spain*

(2) *Instituto de Biología Molecular y Celular, Universidad Miguel Hernández, Elche, Spain.*

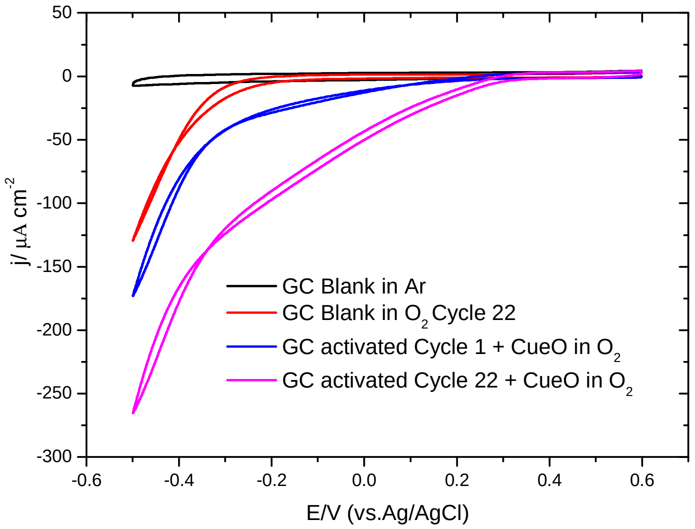

Figure S1. Cyclic voltammogram for CueO immobilized on an activated (during 22 cycles) bare GC in an O_2 saturated PBS, pH 6.5. Blue line: 1st cycle. Pink line: 22nd cycle. GC blank in Ar atmosphere (black line). GC blank in O_2 atmosphere (cycle 22) (Red line). Scan rate: 20 mVs^{-1}.

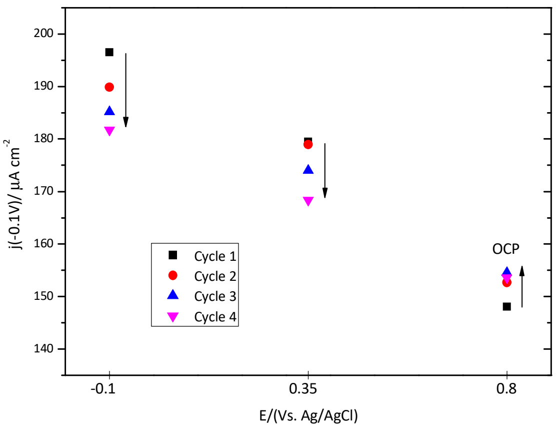

Figure S2. Data for the O_2 reduction catalytic density (for the first four cycles, cycle 1 (black square), cycle 2 (red circle), cycle 3 (blue up-triangle) and cycle 4 (pink down-triangle) after protein submission to OCP, 0.6, 0.35 and -0.1 V potential values.

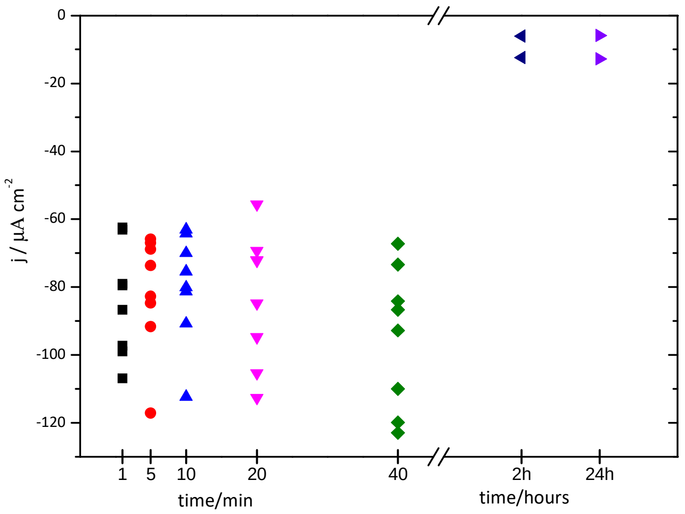

Figure S3. CueO current density dependence with immobilization time on Au(111). O_2 saturated sodium PBS, pH=6.5. Data were collected in the steady curve for a potential value of 0.2 V.

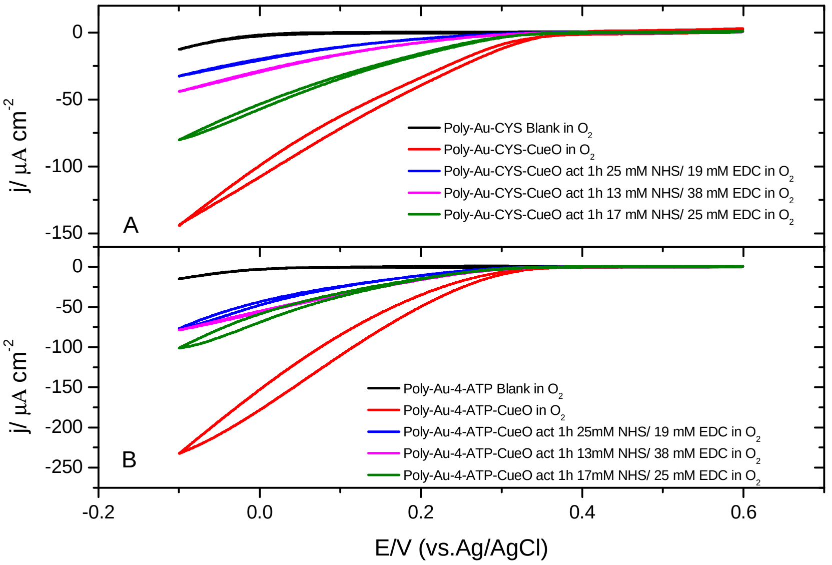

Figure S4. Cyclic voltammograms for CueO activated in presence of different NHS/EDC mixture solutions: blue line (25 mM NHS/ 19 mM de EDC), pink line (13 mM NHS / 38 mM de EDC), green line (17 mM NHS/ 25 mM de EDC). Black line: Poly-Au-CYS blank (A) and (B) Poly-Au-4-ATP blank (B). Red line: Poly-Au-CYS-CueO (A) Poly-Au-4-ATP-CueO (B). O_2 saturated sodium PBS. Scan rate: 20 mVs^{-1}.

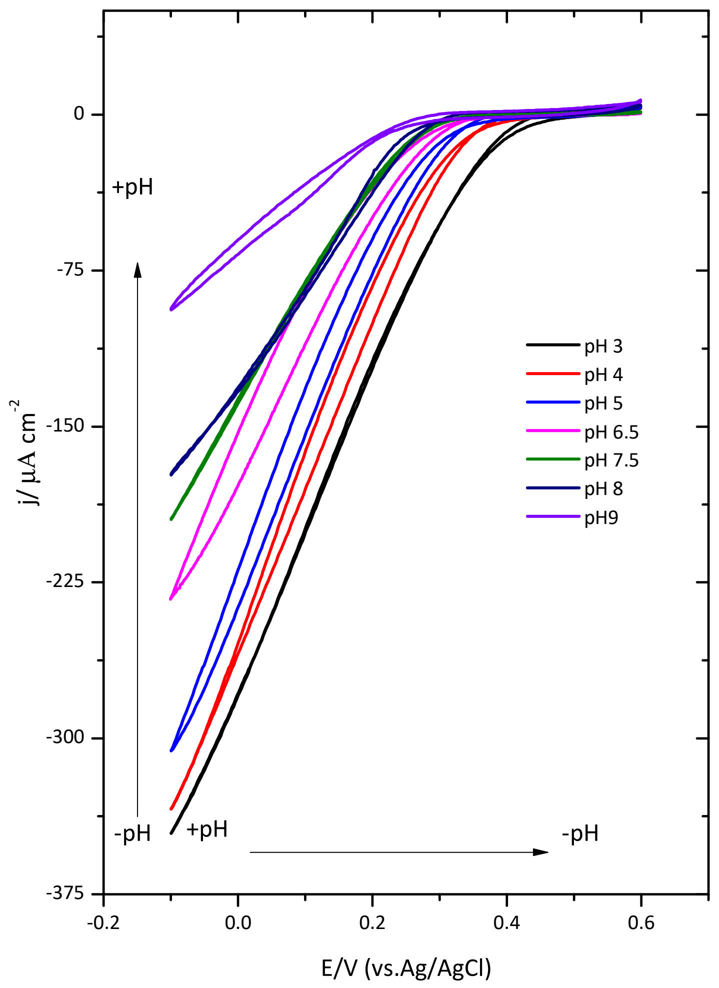

Figure S5. CueO behavior over Au(111) modified with 4-ATP in an O_2 solution at different pHs values between 3 and 9. Scan rate: 20 mVs^{-1}.

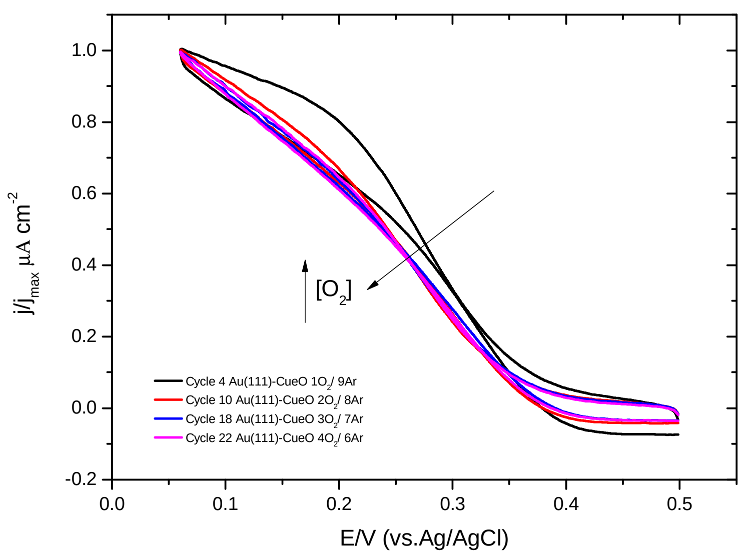

Figure S6. Cyclic voltammogram for the O_2 reduction normalized current densities.

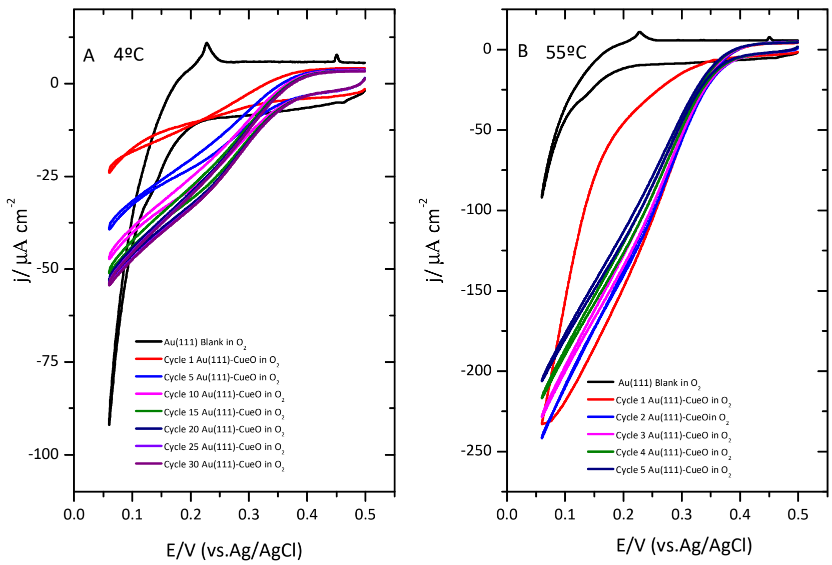

Figure S7. Monitorization of the CueO cyclic voltammograms measured at two different temperatures: (A) 4ºC and B) 55ºC. Scan rate: 20 mVs^{-1}.

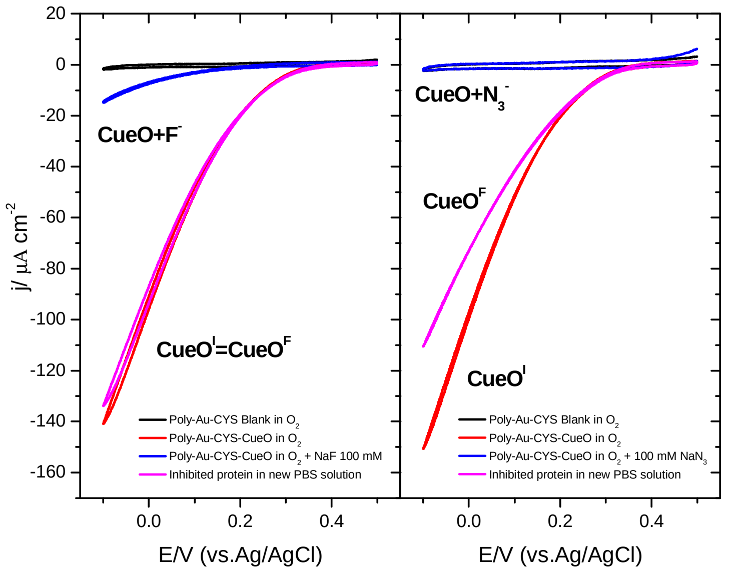

Figure S8. Reversibility of the CueO inhibition process. A) F^- inhibition reversibility process. Cell 1) Poly-Au-CYS blank (black line) and maximum ORR current density for a poly-Au-CYS-CueO modified electrode in absence of inhibitors (red line). Cell 2) ORR current density for a poly-Au-CYS-CueO modified electrode in presence of 100 mM NaF or NaN_3 concentration (blue line). Transference of the inhibited protein modified electrode to the Cell 1) maximum ORR current density for the inhibited poly-Au-CYS-CueO modified electrode in new PBS solution without containing inhibitors (pink line). Scan rate: 2 mVs^{-1}.

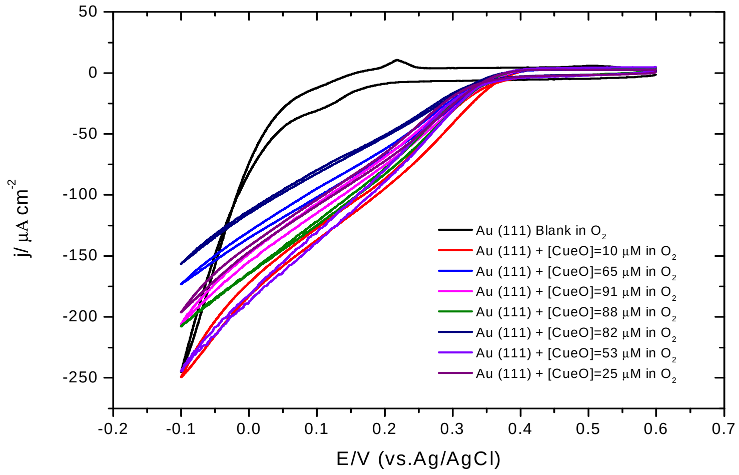

Figure S9. Dependence of the CueO cyclic voltammetry ORR catalysis with the protein concentration. Scan rate: 20 mVs^{-1}.
